# SARS-CoV-2, Mannerism, Marten, Mink, and Man

**DOI:** 10.3201/eid2707.AC2707

**Published:** 2021-07

**Authors:** Mark Swancutt, Terence Chorba

**Affiliations:** Fulton County Board of Health, Atlanta, Georgia, USA (M. Swancutt);; Centers for Disease Control and Prevention, Atlanta (T. Chorba)

**Keywords:** art science connection, emerging infectious diseases, art and medicine, about the cover, Portrait of Antea, Girolamo Francesco Maria Mazzola, Parmigianino, SARS-CoV-2, Mannerism, Marten, Mink, and Man, mustelids, furs, severe acute respiratory syndrome coronavirus 2, SARS-CoV2, coronaviruses, viruses, coronavirus disease, COVID-19, respiratory infections, spillover events, zoonoses, One Health

**Figure:**
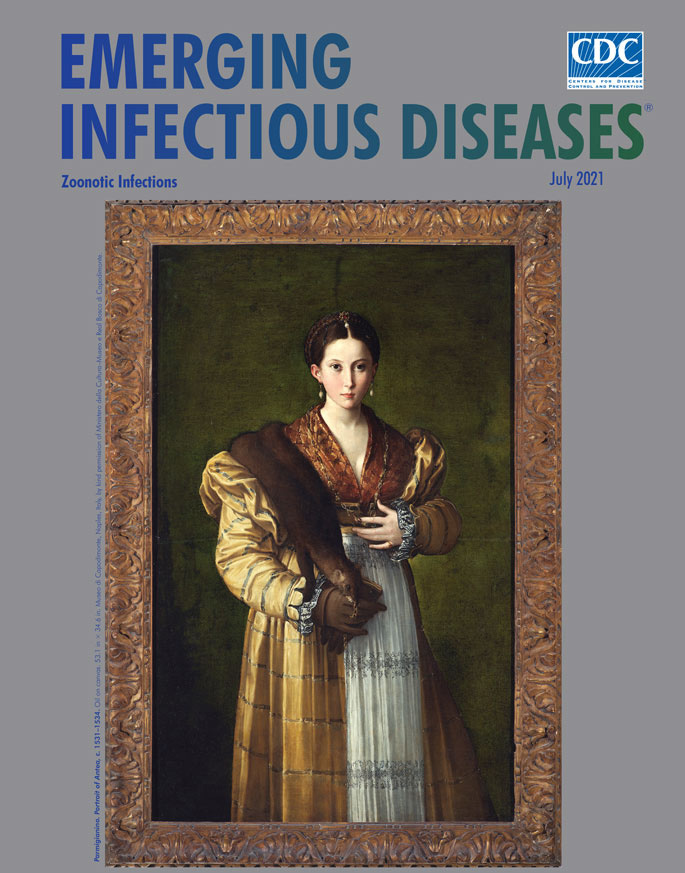
**Parmigianino. Portrait of Antea, c. 1531‒1534. Oil on canvas.** 53.1 in × 34.6 in. Museo di Capodimonte, Naples, Italy. https://en.wikipedia.org/wiki/Antea_(Parmigianino)#/media/File:Parmigianino_007.jpg

Girolamo Francesco Maria Mazzola (1503–1540), known as Parmigianino (the little one from Parma), was a painter and printmaker. He was born into a family of painters in Parma, in what was then the Duchy of Milan, and died in Cremona, in northern Italy. He was among the first and most prominent artists of the Mannerist school of art of the Late Renaissance that came after the High Renaissance. The High Renaissance is known for the works of Raphael and the early works of Michelangelo, and its art is treasured for perfection of elegance and qualities of balance, proportion, and absoluast, in Mannerism, compositions are said to be exaggerated, almost to a fault, as a reaction to the art of the High Renaissance or perhaps as an attempt to improve on it. In general, it is characterized by realist but decorous art in which there is subtle, unsettling elongation of figures with stylized features and poses.

Among Parmigianino’s more renowned works is a portrait painting from 1531‒1534 of a young woman, Antea, which appears on the cover of this month’s journal. It is unknown if the painting is an image of a specific person or a stylized composition. We behold a beautiful young woman facing forward, with elongated bodily proportions, as is characteristic of Mannerist works. With a small oval head set upon a large torso and with wide shoulders, her arms and hands appear exaggerated in size, as if the parts of her body were proportioned for different paintings. Yet despite these physical disproportions, the viewer has an impression of great beauty, a testimony to the subtlety and success of this Mannerist portrait. Contributing to the incongruousness of the image of this young lady, we also note that she is clad in the garments of an older woman, or a woman of an age with which we more commonly associate wealth; Antea is bejeweled, fingering a gold chain with her left hand, and dressed luxuriously. Among her layers of garments are a gold blouse with a fine white apron, a detailed gold satin dress, leather gloves, and a marten fur stole complete with the animal’s head, on which the nose is pierced with a ring still attached to a chain.

For millennia, people have worn animal furs, first for survival and protection, and later for fashion. In European populations, the wearing of furs of Mustelidae, a carnivorous mammalian family, has been popular. Among the many mustelids are wolverines, weasels (including ermines), otters, badgers, martens (including sables), ferrets, and minks. Since the early Middle Ages, the quest for furs of these animals have played a role in expansion efforts of European nations into areas where fur-bearing mustelids were populous, including Russia’s expansion into Siberia and the expansions of France and England into North America. More recently, Mustelidae fur farming, principally of mink, has become widely practiced in North America and Europe.

We are currently in the midst of a global pandemic caused by SARS-CoV-2, a *Betacoronavirus* related to coronaviruses that exist in Chinese horseshoe bats. SARS-CoV-2 spilled over as a zoonosis, perhaps through an intermediate animal, into humans in Hubei, China, a circumstance that was first identified in December 2019. As of 18 April 2021, there were more than 141 million cases of SARS-CoV-2 infection and more than 3 million deaths in humans worldwide. SARS-CoV-2 has also naturally spread as a zoonosis from humans to dogs, domestic cats, large cats in zoos and sanctuaries, gorillas in zoos, and farmed mink, and recently, to pet ferrets. Recent experimental research has shown that many mammals can be infected with the virus, including cats, dogs, bank voles, deer mice, fruit bats, ferrets, hamsters, mink, skunks, pigs, rabbits, raccoon dogs, tree shrews, white-tailed deer, rhesus macaques, and cynomolgus macaques.

Extensive spillback of SARS-CoV-2 from humans to mink on mink farms was first reported in April 2020 in the Netherlands. Mink-to-human transmission has been documented in the Netherlands, Denmark, and Poland and is suspected to have occurred in the United States. In January 2021, a combined report of the Food and Agriculture Organization of the United Nations, the World Organisation for Animal Health (OIE), and the World Health Organization presented data from OIE and other sources from 36 countries with mink farming industries and documented wide-spread virus transmission, in both Europe and North America. Because of concerns that mink farm popu-lations could serve as a reservoir for ongoing coro-naviruses transmission and result in development of mutations that would undermine the effectiveness of SARS-CoV-2 vaccines, large-scale culling of these ani-mals has been pursued by Denmark, the Netherlands, and Spain. Coronavirus variants have been identified in mink farms, including one with three amino-acid changes and two deletions in the spike protein. The escape of mink from farms into the wild is quite common; in one Danish region, 79% of wild mink have been found to be escapees. Transfer of phylogenetically related SARS-CoV-2 from farmed mink into wild mink was first reported in Utah in December 2020. As with any zoonotic pathogen, the transmission of SARS-CoV-2 between animals and people emphasizes the importance of the One Health concept in the global approach to diseases that affect people and animals living in our shared environment.

Just as aspects of the figures in Mannerist paintings often need explanation that can be complicated, difficult, and anxiety-provoking, the issues surrounding fur farming for fashion, potential exchange of SARS-CoV-2 between human populations and various animal species, and the massive culling of the commercial mink population are also complex and controversial. However, it appears that in addition to all the human-human contact prevention measures that will be needed to control and eliminate SARS-CoV-2 transmission, interventions that reduce contacts of humans or domestic (or farmed) animals with bats or other susceptible wild animals will be needed to avert future spillover with pandemic potential. Like the rat-borne Black Plague of the mid-14th century that ravaged the Italian States where Parmigianino executed his magnificent portrayal of Antea with her luxurious mustelid stole a century-and-half later, the current COVID-19 pandemic underscores again how we share pathogens with other species. With more than 1 million deaths and 1 billion infections attributed annually to zoonotic infections before the SARS-CoV-2 pandemic, we are challenged to assess and mitigate the potential risks of SARS-CoV-2 transmission posed by fur farms, both to humans and to other animals. These are but two facets of the multifaceted public health goal of One Health, noted by CDC, “of achieving optimal health outcomes recognizing the interconnection between people, animals, plants, and their shared environment.”
